# iNKT Cells Are Responsible for the Apoptotic Reduction of Basophils That Mediate Th2 Immune Responses Elicited by Papain in Mice Following γPGA Stimulation

**DOI:** 10.1371/journal.pone.0152189

**Published:** 2016-04-06

**Authors:** Hyun Jung Park, Sung Won Lee, Se-Ho Park, Seokmann Hong

**Affiliations:** 1 Department of Bioscience and Biotechnology, Institute of Anticancer Medicine Development, Sejong University, Seoul, 143–747, Korea; 2 School of Life Sciences and Biotechnology, Korea University, Seoul, 136–701, Korea; INEM, FRANCE

## Abstract

Recent studies have demonstrated that *Bacillus subtilis*-derived poly-gamma glutamic acid (γPGA) treatment suppresses the development of allergic diseases such as atopic dermatitis (AD). Although basophils, an innate immune cell, are known to play critical roles in allergic immune responses and repeated long-term administration of γPGA results in decreased splenic basophils in an AD murine model, the underlying mechanisms by which γPGA regulates basophil frequency remain unclear. To investigate how γPGA modulates basophils, we employed basophil-mediated Th2 induction *in vivo* model elicited by the allergen papain protease. Repeated injection of γPGA reduced the abundance of basophils and their production of IL4 in mice, consistent with our previous study using NC/Nga AD model mice. The depletion of basophils by a single injection of γPGA was dependent on the TLR4/DC/IL12 axis. CD1d-dependent Vα14 TCR invariant natural killer T (iNKT) cells are known to regulate a variety of immune responses, such as allergy. Because iNKT cell activation is highly sensitive to IL12 produced by DCs, we evaluated whether the effect of γPGA on basophils is mediated by iNKT cell activation. We found that *in vivo* γPGA treatment did not induce the reduction of basophils in iNKT cell-deficient CD1d KO mice, suggesting the critical role of iNKT cells in γPGA-mediated basophil depletion at the early time points. Furthermore, increased apoptotic basophil reduction triggered by iNKT cells upon γPGA stimulation was mainly attributed to Th1 cytokines such as IFNγ and TNFα, consequently resulting in inhibition of papain-induced Th2 differentiation via diminishing basophil-derived IL4. Taken together, our results clearly demonstrate that γPGA-induced iNKT cell polarization toward the Th1 phenotype induces apoptotic basophil depletion, leading to the suppression of Th2 immune responses. Thus, elucidation of the crosstalk between innate immune cells will contribute to the design and development of new therapeutics for Th2-mediated immune diseases such as AD.

## Introduction

CD4^+^ T cells can be divided into two main subsets (Th1 and Th2) based on their cytokine production: Th1 cells produce IFNγ, IL2, and TNFα/β, whereas Th2 cells produce IL4, IL5, IL10, and IL13. The Th1/Th2 balance is remarkably important for maintaining immune homeostasis [1]; when this balance is broken, Th1-biased immune responses lead to autoimmune conditions such as EAE and type I diabetes, whereas Th2 predominance can result in allergic disorders such as asthma and AD. Because the antagonization of Th2 cell function by Th1 cells is believed to protect against Th2-mediated allergic immune responses, controlling Th2 effectors through the recruitment of Th1 cells is considered to be a rational strategy for decreasing allergic pathogenesis. However, some previous reports have demonstrated that Ag-specific Th1 cells alone are not effective at inhibiting Th2 cell development or preventing Th2-induced airway hypersensitivity, suggesting the requirement of additional factors modulating Th2 immune responses [2, 3].

Because dendritic cells (DCs) are essential antigen-presenting cells (APCs) that function in the differentiation of naive CD4^+^ T cells into T cell subsets via polarizing cytokines, DCs are one of the main targets for suppressing allergen-specific Th2 immune responses. DC-based Th2 induction was previously considered to depend on the differential expression of B7-1 (CD80)/B7-2 (CD86) [4], the production of OX40 ligand by thymic stromal lymphopoietin (TSLP) stimulation [5], and the secretion of TSLP [6]. A recent paper provides evidence that Kruppel-like factor-4 (KLF4) is a key transcriptional regulator in IRF4-expressing conventional DCs (cDCs) to promote Th2 immune responses [7]. The identification of APCs responsible for producing IL4 has remained elusive, but recent studies have suggested that basophils, one of innate effector cells involved in initiating allergic immune responses, can induce Th2 differentiation in response to a protease allergen such as papain through the production of IL4 and/or TSLP [8] and can also act as APCs to promote Th2 immune responses [9, 10]. These findings provide fundamental information for designing a better strategy for the treatment of allergic diseases via basophil-based immune modulation.

Among NKT cells expressing NK1.1, invariant NKT (iNKT) cells are well characterized by their expression of an invariant TCR encoded by *Vα14-Jα18* in mice and by *Vα24-Jα18* in humans and are among the innate lymphocytes that recognize lipid/glycolipid antigens presented by the MHC I-like molecule CD1d. In addition, iNKT cells can induce direct cytotoxicity against tumor cells via the secretion of perforin/granzyme B and the expression of Fas/FasL. As iNKT cells are considered to be multifunctional cells based on their abilities to produce both Th1 (e.g., TNFα, IFNγ, and IL2) and Th2 (e.g., IL4, IL5, IL10, and IL13) cytokines, iNKT cells have been suggested to play either protective or pathogenic roles in different pathogenic conditions [11]. In particular, IFNγ produced by iNKT cells has protective effects against allergic reactions such as asthma and rhinitis [12, 13]. Furthermore, the IFNγ produced by iNKT cells increases IL12 secretion by DCs [14]; in turn, upregulated IL12 production by DCs can trigger iNKT cells to secrete IFNγ [15], indicating that such a positive feedback loop between NKT cells and DCs is required for optimal Th1 immune responses.

Poly-γ-glutamate (γPGA), an unusual anionic polypeptide in which D- and/or L-glutamate is polymerized via γ-amide linkages, is a safe and edible biomaterial naturally synthesized by *Bacillus subtilis* isolated from chungkookjang [16]. γPGA promotes Th1 differentiation through increased IL12p40 production by DCs [17] and IFNγ production by natural killer (NK) cells in a TLR4-dependent manner, and such an enhanced Th1 response is associated with increased antitumor effects [18, 19]. Depending on their expression of NK1.1, DCs can be divided into NK1.1^+^ DCs (called NKDCs) and NK1.1^-^ cDCs. Recently, we revealed that DCs differentially produce Th1-type cytokines (IFNγ and IL12) upon stimulation with γPGA, with preferential production of IFNγ by NKDCs and predominant IL12 secretion by cDCs [20]. Moreover, recent studies have shown that increased Th1 immune responses after γPGA injection suppress the development of Th2-dominant diseases, including asthma [21] and AD [22, 23].

In this study, we investigated the *in vivo* suppressive effects of γPGA on basophil-mediated Th2 immune responses as a result of papain treatment in mice. We found that γPGA suppressed papain-induced Th2-polarized immune responses via both DC-derived IL12- and iNKT cell-dependent mechanisms. We demonstrate for the first time that iNKT cells play a major role in the γPGA-mediated suppression of Th2 immune responses through the production of Th1-type cytokines such as IFNγ and through the induction of apoptosis in basophils identified as an early source of IL4.

## Materials and Methods

### Mice

Wild-type (WT) C57BL/6 (B6), WT Balb/c, C3H/HeN (TLR4-WT), C3H/HeJ (TLR4-mutant), *lpr/lpr* (Fas mutant), and *gld/gld* (FasL mutant) mice were purchased from Jung Ang Lab Animal Inc. (Seoul, Korea). The C3H/HeN and C3H/HeJ mice were of the C3H background and the *lpr/lpr* and *gld/gld* mice were of the B6 background. CD1d knockout (KO) and Vα14 TCR transgenic (Tg) mice were provided by Dr. A. Bendelac (University of Chicago, IL, USA). Jα18 KO mice were gifts from Dr. M. Taniguchi (RIKEN, Yokohama, Japan). The CD1d KO, Vα14 TCR Tg, and Jα18 KO mice were of the B6 background. WT NC/Nga mice were purchased from Jung Ang Lab Animal Inc. (Seoul, Korea). The CD1d KO and Vα14 TCR Tg mice were backcrossed to NC/Nga mice for more than eleven generations. The IL4/GFP reporter (4get) and IL12p35 KO mice were kindly provided by Dr. R. Locksley (University of California at San Francisco, CA, USA). The 4get and IL12p35 KO mice were of the Balb/c and B6 backgrounds, respectively. CD11c-diphtheria toxin receptor (DTR) Tg B6 mice were obtained from Dr. E. Choi (Seoul National University, Seoul, Korea). All the mice were maintained at Sejong University, and used at 6–12 weeks of age for experiments. They were maintained on a 12-hour light/12-hour dark cycle in a temperature-controlled barrier facility with free access to food and water. These mice were fed a γ-irradiated sterile diet and autoclaved tap water. In this study, age- and sex-matched mice were used for all the experiments. The animal experiments were approved by the Institutional Animal Care and Use Committee at Sejong University (SJ-20130801).

### Reagents

γPGA was purchased from Bioleaders (Daejeon, Korea), dissolved in a neutral pH buffer, diluted in PBS, and utilized at a final concentration of 10 mg/ml. Diphtheria toxin (DT) derived from *E*. *coli* (serotype 0111:B4) was purchased from Sigma-Aldrich (St. Louis, MO, USA). Recombinant murine IL3 and IL4 were purchased from R&D systems (Minneapolis, MN, USA). For *in vitro* stimulation, IL3 and IL4 were used at a concentration of 20 ng/ml and 5 ng/ml, respectively. Lipopolysaccharide (LPS) derived from *E*. *coli* (serotype 0111:B4) was purchased from Sigma-Aldrich (St. Louis, MO, USA).

### Cell isolation by magnetic activated cell sorting (MACS) and culture

A single-cell suspension of splenocytes was prepared and resuspended in RPMI complete medium consisting of RPMI 1640 (Gibco BRL, USA) medium supplemented with 10% FBS, 10 mM HEPES, 2 mM L-glutamine, 100 units/mL penicillin-streptomycin, and 5 mM 2-mercaptoethanol. NK and iNKT cells were enriched using the NK cell isolation kit II and NK1.1 iNKT cell isolation kit (Miltenyi Biotech, Bergisch Gladbach, Germany) following the manufacturer’s instructions, respectively. The NK population was >87% pure and NKT population was >91% pure after MACS. In addition, for the preparation of CD11c^+^ total DCs, whole splenocytes from WT B6 mice were stained with anti-CD11c monoclonal antibody (mAb) for MACS and enriched for CD11c^+^ DCs by positive selection. The DC population was >95% after MACS. Bone marrow-derived basophils (BM basophils) were separated as follows: IL3-cultured BM cells were stained with biotin-conjugated anti-CD49b (clone DX5) mAbs, and then DX5^+^ cells were positively selected using anti-biotin MACS beads. The basophil population was >92% after MACS.

### Flow cytometry

The following mAbs from BD Biosciences were used: fluorescein isothiocyanate (FITC)- or phycoerythrin (PE)-Cy7- or allophycocyanin (APC)-conjugated anti-CD3ε (clone 145-2C11); PE- or APC-conjugated anti-NK1.1 (clone PK-136); PE-Cy7-conjugated anti-CD69 (clone H1.2.F3); biotin-conjugated anti-CD49b (clone DX5); APC-conjugated anti-CD19 (clone ID3); PE-Cy7-conjugated anti-CD4 (clone RM4-5); PE-Cy7- or APC-conjugated anti-CD11c (clone HL3); biotin-conjugated anti-CD86 (clone GL1); PE-conjugated anti-MHC II (clone M5/114.15.2); PE-conjugated anti-Fas (clone Jo2); PE-conjugated anti-FasL (clone NOK-1); PE-conjugated anti-TLR4 (clone MTS510); biotin-conjugated anti-CD212 (IL12 receptor β1) (clone 114); PE-conjugated anti-TNFα (clone MP6-XT22); PE-conjugated anti-IFNγ (clone XMG1.2); PE-conjugated anti-IL4 (clone BVD6-24G2); PE-conjugated anti-IL12p40 (clone C15.6); and PE-conjugated anti-IgG1 (κ isotype control) (clone R3-34). The following mAbs from eBioscience were used: FITC- or PE-conjugated anti-FcεRI (clone MAR-1) and PE-conjugated anti-CD119 (IFNγ receptor 1) (clone 2E2). The following mAbs from BioLegend were used: PE-conjugated anti-CD120a (TNF receptor type 1) (clone 55R-286). To perform surface staining, cells were harvested, washed twice with cold 0.5% BSA-containing PBS (FACS buffer), and then were incubated with anti-CD16/CD32 mAbs on ice for 10 min for blocking Fc receptors. Subsequently these cells were stained with fluorescence-labeled mAbs. Flow cytometric data were acquired using a FACSCalibur flow cytometer (Becton Dickson, San Jose, CA, USA) and analyzed using FlowJo software (Tree Star Inc., Ashland, OR, USA).

### Intracellular cytokine staining

Splenocytes were purified from either PBS- or γPGA-injected mice. To perform intracellular staining, splenocytes were incubated with brefeldin A, an intracellular protein transport inhibitor (10 μg/ml), in RPMI medium for 2 hrs at 37°C. The cells were stained for cell surface markers, fixed with 4% PFA, washed once with cold FACS buffer, and permeabilized with 0.5% saponin. The permeabilized cells were then stained for an additional 30 min at room temperature with the indicated mAbs (PE-conjugated anti-IFNγ, PE-conjugated anti-TNFα, PE-conjugated anti-IL12p40, PE-conjugated anti-IL4, or PE-conjugated isotype control rat IgG mAbs). More than 5,000 cells per sample were acquired using a FACSCalibur and analyzed with the FlowJo software package.

### Generation of BM basophils

BM basophils were generated from the bone marrow cells of mice, as previously described. Briefly, bone marrow cells from femurs and tibiae of the indicated mice were flushed with complete RPMI 1640 medium. After the removal of red blood cells (RBCs) using ACK lysis buffer (0.15 M NH_4_Cl, 10 mM KHCO_3_, and 2 mM EDTA), the bone marrow cells were washed with PBS and cultured at a concentration of 1 x 10^6^ cells/ml in complete RPMI 1640 medium supplemented with recombinant mouse IL3 (20 ng/ml) for 10 days in 24-well tissue culture plates.

### *In vivo* stimulation

**Fig 1:**
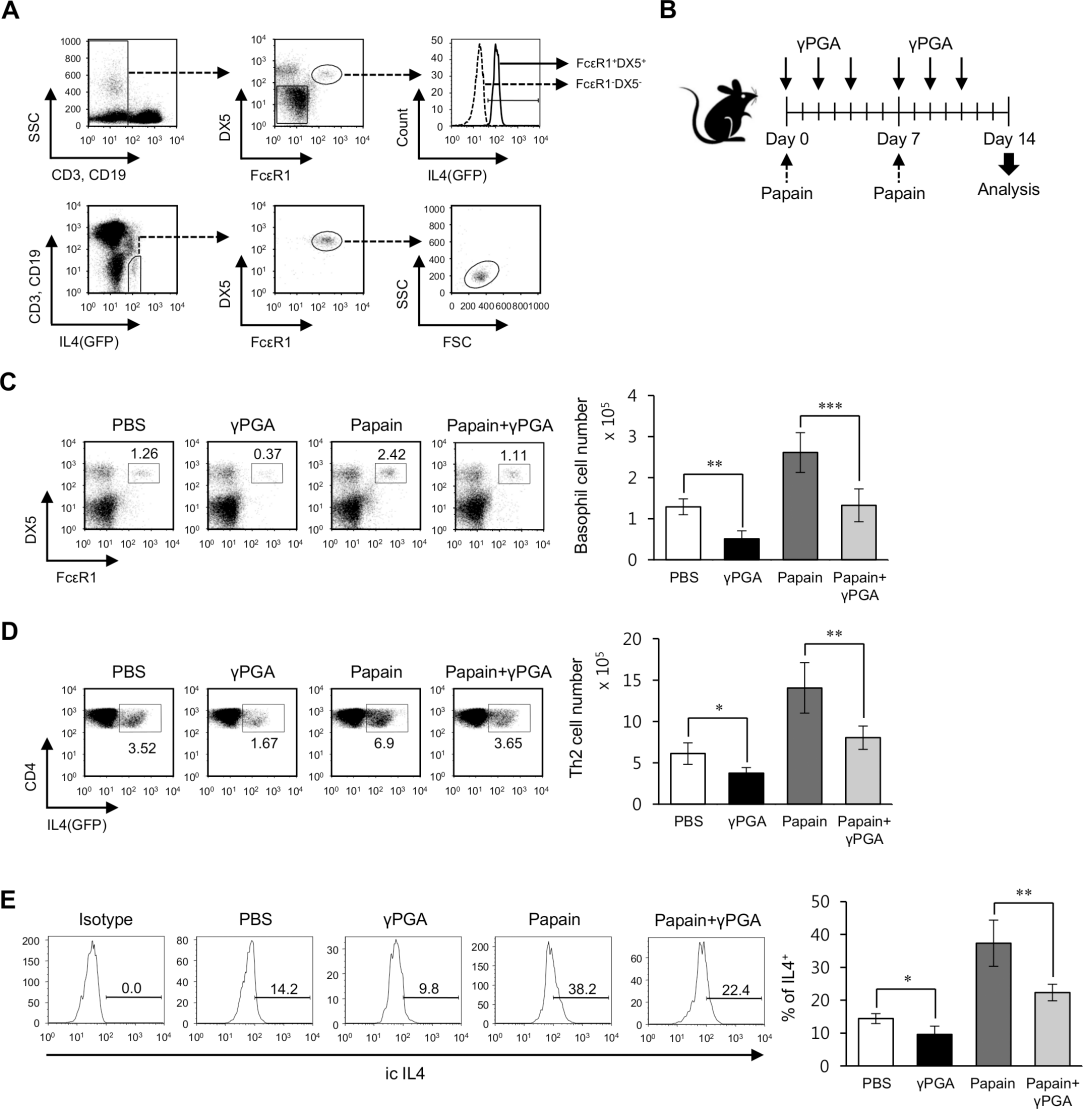
Administration of γPGA attenuates Th2 immune responses elicited by the cysteine protease papain through a decrease in basophils. (A) Total splenocytes were isolated from 4get Balb/c mice. IL4(GFP) expression in basophils (FcεRI^+^DX5^+^) and non-basophils (FcεRI^-^DX5^-^) was assessed on CD3ε^-^CD19^-^ gated populations using flow cytometry (upper panel). The basophil population was calculated by gating (CD3ε^-^CD19^-^IL4(GFP)^+^), as observed in the lower panel. One representative result is shown (n = 3 per group in the experiment). (B) 4get Balb/c mice were immunized i.p. with 500 μg papain once a week for 2 weeks. Either PBS or γPGA (2 mg) was i.p. injected into PBS- or papain-treated mice a total of 6 times during 2 weeks. Splenocytes were analyzed at day 14, as observed in B. (C) The frequency of basophils (FcεRI^+^DX5^+^) among lineage-negative cells (CD3ε^-^CD19^-^) of total splenocytes was plotted on day 14 after immunization (left panel). The absolute number of basophils was determined (right panels). The mean values ± SD (n = 4 per group in the experiment; Student’s t-test; **P<0.01, ***P<0.001) are shown. (D) IL4(GFP) expression was measured in CD4^+^ T cells (CD3ε^+^CD4^+^) of the spleen using flow cytometry. The mean values ± SD (n = 4 per group in the experiment; Student’s t-test; *P<0.05, **P<0.01) are shown. (E) WT Balb/c mice were immunized i.p. with 500 μg papain once a week for 2 weeks. Either PBS or γPGA (2 mg) was i.p. injected into PBS- or papain-treated mice a total of 6 times during 2 weeks. Intracellular IL4 production in splenic basophils (CD3ε^-^CD19^-^FcεRI^+^DX5^+^) was assessed via flow cytometry on day 14 after first immunization. The mean values ± SD (n = 3 per group in the experiment; Student’s t-test; *P<0.05, **P<0.01) are shown.

Mice were immunized intraperitoneally (i.p.) with 500 μg papain once a week for 2 weeks. Either γPGA (2 mg/injection) or PBS alone was i.p. administered into PBS- or papain-treated mice a total of 6 times during 2 weeks (Fig 1B). Fourteen days later, the frequency of basophils and the polarization of Th2 cells were assessed in splenocytes from the indicated mice.

### *In vitro* CD4^+^ T cell differentiation

Naive CD4^+^ T cells from Jα18 KO B6 mice were separated with the CD4^+^CD62L^+^ T cell isolation kit II (Miltenyi Biotech, Bergisch Gladbach, Germany), following the manufacturer’s instructions. The naive CD4^+^ T cells were >95% pure after MACS. These naive CD4^+^ T cells (1 × 10^6^ cell/ml) were incubated with a combination of basophils (6 × 10^4^ cells/well), papain (25 μg/ml), rIL4 (10 ng/ml), or neutralizing anti-IL4 mAbs (5 μg/ml) in a 96-well plate pre-coated with anti-CD3 (10 μg/ml) and anti-CD28 (1 μg/ml) mAbs in the absence or presence of iNKT cells (1.2 × 10^6^ cells/well) purified from Vα14 TCR Tg B6 mice treated with either PBS or γPGA for 5 days.

### Statistical analysis

Statistical significance was determined using Excel (Microsoft, USA). Student’s t-test was performed for the comparison of two groups. *P<0.05, **P<0.01, and ***P<0.001 were considered to be significant in the Student’s t-test. Two-way ANOVA analysis was carried out using the VassarStats (http://faculty.vassar.edu/lowry/VassarStats.html). ^#^P<0.05, ^##^P<0.01, and ^###^P<0.001 were considered to be significant in the two-way ANOVA.

## Results

### *In vivo* administration of γPGA attenuates Th2 immune responses elicited by the cysteine protease papain via basophil reduction

Previously, we found that the long-term administration of γPGA prevented the progression of AD in NC/Nga AD model mice through a dramatic decrease in splenic basophils, which are one of key players in allergic immune responses [22]. However, the cellular mechanism by which γPGA decreases the abundance of basophils has remained unclear. To examine the suppressive effects of γPGA on basophil-mediated Th2 immune responses, we employed a papain-induced Th2 model that has been shown to be entirely dependent on the presence of basophils [9]. Consistent with previous reports [9, 24], we confirmed in the 4get (IL4 cytokine reporter) mouse model that basophils (FcεRI^+^DX5^+^CD3ε^-^CD19^-^) express high levels of IL4(GFP) and vice versa, i.e., the IL4(GFP)^high^ population in non-B/non-T cells is largely composed of FcεRI^+^DX5^+^ cells (Fig 1A). Following i.p. injection of papain into 4get Balb/c mice as shown in Fig 1B, the frequency of splenic basophils was significantly increased by approximately 200%, whereas *in vivo* γPGA injection almost completely blocked the increased number of basophils due to papain stimulation. Even a single treatment of γPGA could significantly induce the reduction of basophils (Fig 1C). Moreover, papain treatment remarkably elevated the number of IL4(GFP)^+^ Th2 cells by approximately 200%, whereas papain-mediated increase of Th2 cells was inhibited by γPGA injection. Such decrease in Th2 cells was observed when treated with γPGA alone (Fig 1D). In addition, γPGA injection alone suppressed IL4 production by basophils. Furthermore, we found that the increased IL4 production by basophils upon papain stimulation was significantly diminished by γPGA injection (Fig 1E). Taken together, these results provide evidence that the *in vivo* injection of γPGA has a negative influence on basophil-mediated Th2 immune responses elicited by papain treatment.

### DC-derived IL12 is responsible for the reduction in basophils by γPGA stimulation

**Fig 2:**
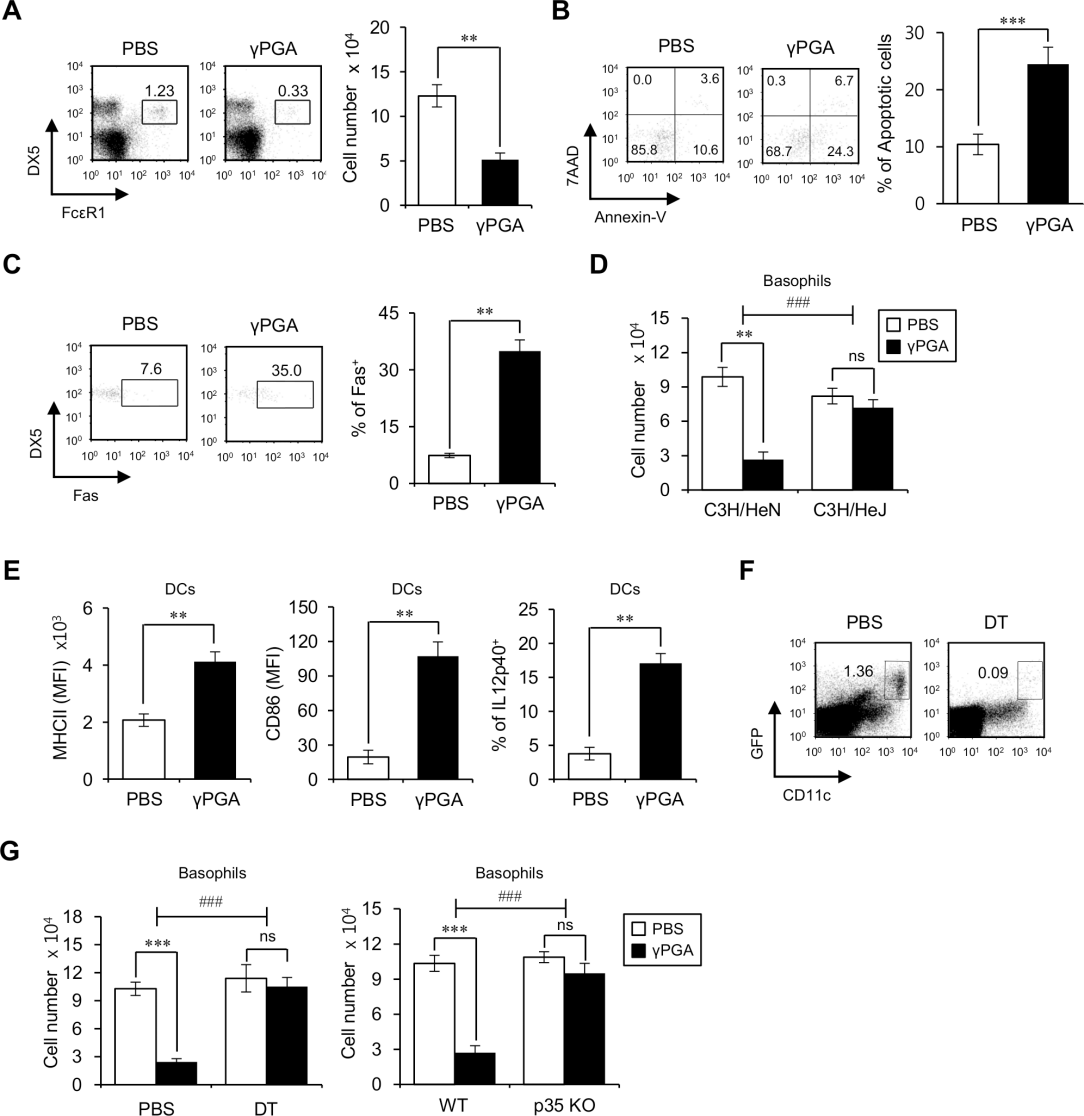
DC-derived IL12 is responsible for the reduction of basophils by γPGA stimulation. (A-B) Splenocytes were prepared from either PBS- or γPGA-injected mice at 16 hrs after treatment. (A) The frequency of basophils (FcεRI^+^DX5^+^) among lineage-negative cells (CD3ε^-^CD19^-^) of total splenocytes was plotted (left panel). The absolute number of basophils was determined (right panels). The means ± SD are shown (n = 3 per group in the experiment; Student’s t-test; **P<0.01). (B) DX5^+^ splenocytes were purified by using anti-biotin MACS after staining total splenocytes with biotin-conjugated anti-DX5 mAb. The frequency of apoptotic cells (annexin-V^+^7AAD^-^) among basophils (FcεRI^+^CD3ε^-^CD19^-^) was assessed by flow cytometry. Left, representative FACS plots; right, summary. The mean values ± SD are shown (n = 4 per group in the experiment; Student’s t-test; ***P<0.001). (C) Expression of Fas on basophils was assessed by flow cytometry. Left, representative FACS plots; right, summary. The mean values ± SD are shown (n = 3 per group in the experiment; Student’s t-test; **P<0.01). (D) Either PBS or γPGA was i.p. injected into C3H/HeN and C3H/HeJ mice and 16 hrs later splenocytes were prepared. The absolute number of basophils was determined. The mean values ± SD are presented (n = 3 per group in the experiment; Student’s t-test; **P<0.01, ***P<0.001). Two-way ANOVA (genotype × treatment) showed an interaction between these two factors (^###^P<0.001). (E) Splenocytes were prepared from either PBS- or γPGA-injected mice at 16 hrs after treatment. Expression of MHC class II/CD86 and intracellular IL12p40 production were analyzed in DCs (CD11c^+^). The means ± SD are shown (n = 3 per group in the experiment; Student’s t-test; **P<0.01). (F) PBS or DT (120 ng/mouse) was used to treat CD11c-DTR Tg B6 mice, and the frequencies of DCs (CD11c^+^GFP^+^) among total splenocytes was plotted 16 hrs later. One representative result is shown (n = 3 per group in the experiment). (G) Either PBS or γPGA was i.p. injected into WT, DT (120 ng/mouse)-treated CD11c-DTR Tg, and IL12p35 KO B6 mice. The absolute number of basophils was determined. The mean values ± SD are shown (n = 3 per group in the experiment; Student’s t-test; ***P<0.001). Two-way ANOVA (genotype × treatment) showed an interaction between these two factors (^###^P<0.001).

*Bacillus subtilis*-derived γPGA is an adjuvant known to promote the expression of T-bet, a key transcription factor for Th1 differentiation, subsequently leading to the development of Th1 cells [17]. Because Th1-dominant immune responses have been shown to inhibit the expansion of basophils and their production of IL4 [25, 26] and to promote basophil apoptosis [27], we investigated whether the *in vivo* injection of γPGA can affect the frequency and activation status of basophils. For this purpose, basophil populations were analyzed from WT B6 mice injected i.p. with γPGA for 16 hrs. We found that a single injection of γPGA significantly decreased the frequency and absolute cell number of splenic basophils (Fig 2A). Because *in vitro* anti-Fas antibody treatment induces basophils to undergo early apoptosis [28], resulting in the increased expression of an apoptosis marker (annexin-V and 7AAD) and a death receptor (Fas) [27], we examined whether the reduction of basophils due to γPGA is associated with early apoptosis. We observed markedly elevated apoptosis (annexin-V^+^7AAD^-^) and Fas expression in the basophils of γPGA-treated mice compared with the controls (Fig 2B and 2C), suggesting that γPGA induces basophil depletion via the apoptotic pathway. It has been reported that Th1 differentiation induced by γPGA is dependent on the TLR4/DC/IL12 axis [17, 19]. Thus, we examined whether TLR4 is responsible for γPGA-mediated basophil reduction using TLR4-mutant C3H/HeJ and TLR4-sufficient C3H/HeN mice; as expected, γPGA treatment did not diminish the basophil population in C3H/HeJ mice unlike in C3H/HeN mice, suggesting that basophil reduction by γPGA is mediated through the TLR4 pathway (Fig 2D). Moreover, because DCs are known to initiate γPGA-mediated immune responses [17, 19], we analyzed the effect of γPGA on DCs. A single *in vivo* injection of γPGA up-regulated IL12 production and the expression of MHC class II molecules and costimulatory molecules such as CD86 in DCs (Fig 2E). Next, to test whether these γPGA-activated DCs can affect basophil reduction, we took advantage of CD11c-DTR Tg B6 mice in which CD11c^+^ DCs can be depleted by a single i.p. injection of DT. Injection of DT (120 ng/mouse) effectively induced the depletion of splenic DCs (CD11c^+^/GFP(CD11c)^+^) (Fig 2F), and we found that basophil depletion due to γPGA injection did not occur in DT-treated CD11c-DTR Tg B6 mice, suggesting that DCs are the main mediator of basophil reduction by γPGA (Fig 2G, left panel). Although basophils constitutively express TLR4 on their cell surfaces (S2 Fig), these results indicate basophil reduction by γPGA was mediated through other TLR4^+^ cells such as DCs rather than via direct TLR4 signaling into basophils.

Moreover, because γPGA induces DCs to produce IL12, we examined whether IL12 produced from DCs plays an important role in basophil reduction. For this purpose, splenocytes from either WT B6 or IL12p35 KO B6 mice were stimulated with γPGA for 16 hrs, and then the extent of basophil reduction was measured by flow cytometry. γPGA treatment did not cause a reduction in basophil number in IL12p35 KO B6 mice, suggesting a critical role for IL12 in the maintenance of the basophil population (Fig 2G, right panel). In addition, the repeated oral administration of γPGA induced basophil reduction comparable to the level of repeated i.p. injection. Furthermore, basophil reduction by a single γPGA injection was almost restored to the original state 5 days post-γPGA treatment (S3 Fig), regardless of oral or i.p. injection. Taken together, these results demonstrate that *in vivo* treatment of γPGA induces a decrease in the basophil population through the TLR4/DC/IL12 axis.

### γPGA-mediated reduction of basophils at the early time points is dependent on CD1d-restricted iNKT cells

**Fig 3:**
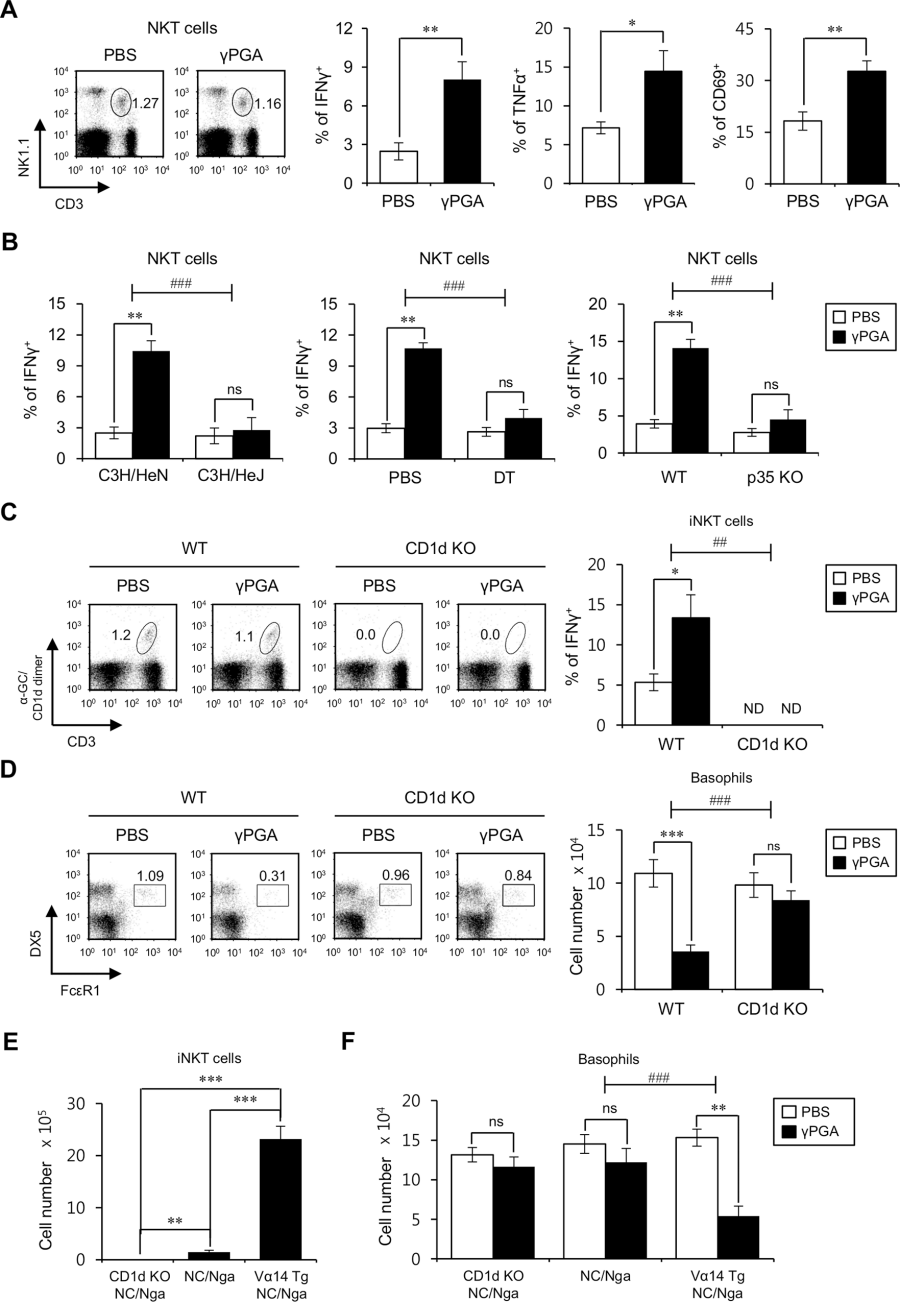
The reduction in basophil number by γPGA stimulation is dependent on CD1d-restricted iNKT cells. (A) Either PBS or γPGA was i.p. injected into WT B6 mice, and splenocytes were isolated 16 hrs later. The frequencies of NKT cells (NK1.1^+^CD3ε^+^) among total splenocytes were plotted. One representative result is shown (left panel). Intracellular IFNγ and TNFα production (middle panel) and CD69 expression (right panel) were analyzed in NKT cells (NK1.1^+^CD3ε^+^). Representative data of three independent experiments are shown (n = 3 per group in the experiment; Student’s t-test; *P<0.05, **P<0.01). (B) Either PBS or γPGA was i.p. injected into WT B6, C3H/HeN, C3H/HeJ, DT (120 ng/mouse)-treated CD11c-DTR Tg B6, and IL12p35 KO B6 mice, and splenocytes were prepared 16 hrs later. Intracellular IFNγ production was analyzed in NKT cells (NK1.1^+^CD3ε^+^). The mean values ± SD are shown (n = 3 per group in the experiment; Student’s t-test; **P<0.01). Two-way ANOVA (genotype × treatment) showed an interaction between these two factors (^###^P<0.001). (C-D) Either PBS or γPGA was i.p. injected into WT and CD1d KO B6 mice, and splenocytes were prepared 16 hrs later. (C) The frequency of iNKT cells (α-GC/CD1d dimer^+^CD3ε^+^) among total splenocytes was plotted (left panel), and intracellular IFNγ production in iNKT cells was determined via flow cytometry (right panels). The mean values ± SD are shown (n = 3 per group in the experiment; Student’s t-test; *P<0.05). Two-way ANOVA (genotype × treatment) showed an interaction between these two factors (^##^P<0.01). (D) The frequency of basophils (FcεRI^+^DX5^+^) among lineage-negative cells (CD3ε^-^CD19^-^) among total splenocytes was plotted (left panel). The absolute number of basophils was determined (right panels). The means ± SD are shown (n = 3 per group in the experiment; Student’s t-test; ***P<0.001). Two-way ANOVA (genotype × treatment) showed an interaction between these two factors (^##^P<0.01). (E-F) Either PBS or γPGA was i.p. injected into WT NC/Nga, CD1d KO NC/Nga, and Vα14 TCR Tg NC/Nga mice and 16 hrs later splenocytes were prepared. (E) The absolute cell number of iNKT cells (α-GC/CD1d dimer^+^CD3ε^+^) was determined. The mean values ± SD are shown (n = 3 per group in the experiment; Student’s t-test; **P<0.01, ***P<0.001) (F) The absolute number of basophils (FcεRI^+^DX5^+^) among lineage-negative cells (CD3ε^-^CD19^-^) was assessed by flow cytometry. The mean values ± SD are shown (n = 3 per group in the experiment; Student’s t-test; **P<0.01). Two-way ANOVA (genotype × treatment) showed an interaction between these two factors (^##^P<0.01).

It has been previously demonstrated that IL12 secreted by DCs can activate iNKT cells and further induce them to produce proinflammatory cytokines [29]. As innate immune cells responsive to IL12, NKT cells display an approximately 8-fold increase in IL12 receptor expression on their surface compared to NK cells (S1 Fig); thus, we next examined whether γPGA activates NKT cells depending on DC-derived IL12, which is essential for NKT cell activation *in vivo*. As a first step, we have confirmed that upon *in vivo* γPGA stimulation, both cytokine production (IFNγ and TNFα) and the expression of activation marker (CD69) were increased in NKT cells (Fig 3A). Interestingly, NKT cells *in vivo* produced lower levels of IFNγ in the absence of TLR4/DC/IL12 signaling compared with the control when stimulated by γPGA (Fig 3B). To examine whether the γPGA-induced decrease in basophils could be affected by iNKT cells, we took advantage of CD1d KO B6 mice, which lack CD3ε^+^αGC/CD1d dimer^+^ iNKT cells (Fig 3C, left panel), and found that γPGA induced IFNγ production in iNKT cells from WT B6, but not from CD1d KO B6 mice (Fig 3C, right panel). We also found that the basophil number was significantly decreased by γPGA stimulation in WT B6 mice but was restored in CD1d KO B6 mice (Fig 3D), suggesting that iNKT cells are one of the key players regulating the basophil population. By employing WT NC/Nga mice, which are known to have fewer NKT cells due to the deletion of Vβ8 genes, we further confirmed the correlation between the presence of iNKT cells and basophil reduction upon *in vivo* γPGA treatment. As expected, a significant decrease in basophils after *in vivo* γPGA treatment was not observed in WT NC/Nga or in CD1d KO NC/Nga mice, in which CD1d-dependent iNKT cells (among the total NKT cell population) are totally deficient. In contrast, introduction of a Vα14 TCR transgene into NC/Nga mice resulted in an increase of iNKT cells, which are critically involved in the reduction of basophils after γPGA injection (Fig 3E and 3F). Therefore, our results demonstrate for the first time that Vα14 iNKT cells are responsible for the reduction of basophils upon *in vivo* γPGA treatment.

### Activation of iNKT cells by γPGA-treated DCs contributes to basophil apoptosis via Th1-type cytokines but not the Fas/FasL pathway

**Fig 4:**
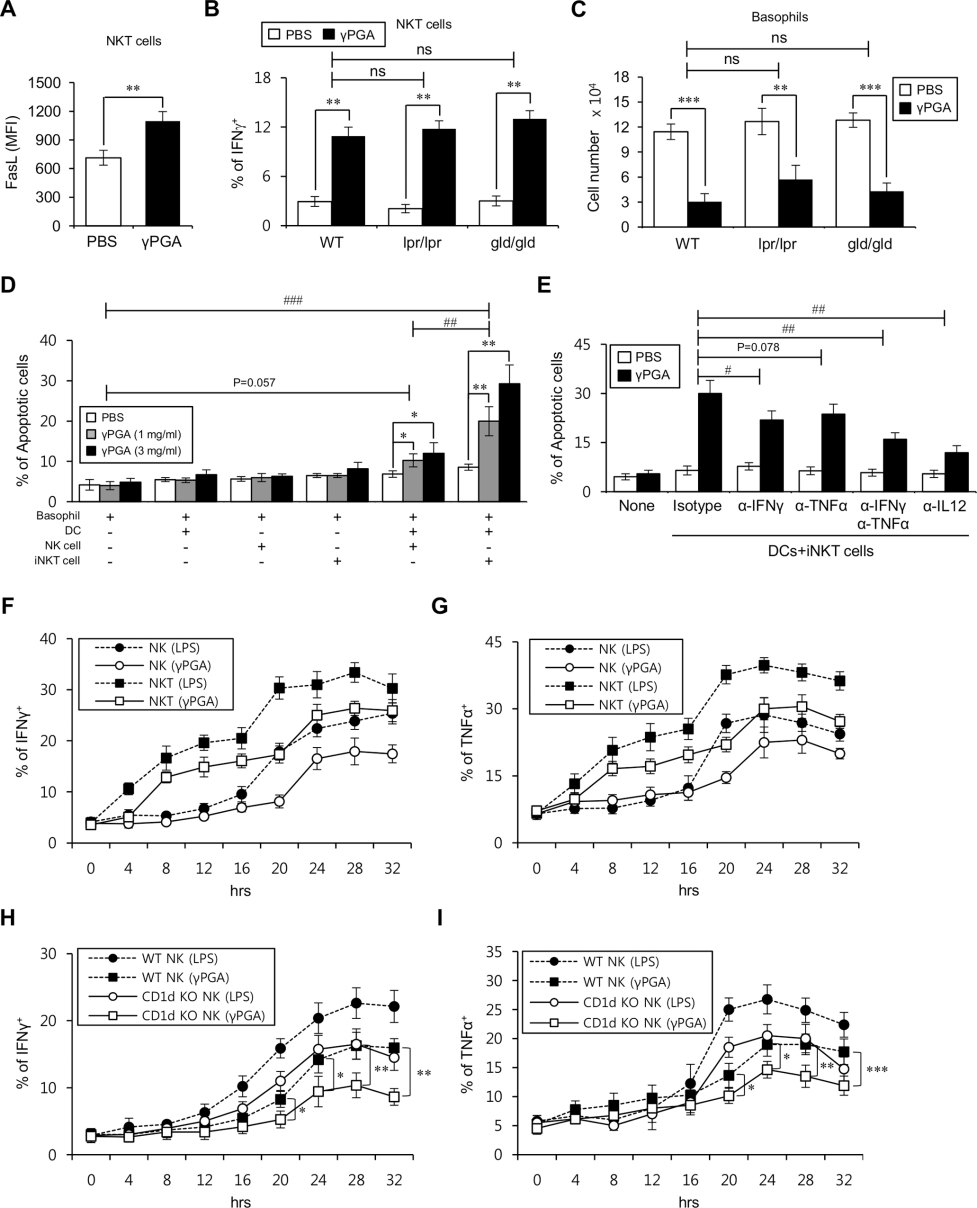
Activated iNKT cells by γPGA-stimulated DC stimulation contributes to basophil apoptosis via cytokines but not the Fas/FasL pathway. (A) Splenocytes were prepared from either PBS- or γPGA-injected mice at 16 hrs after treatment. Expression of FasL was analyzed in NKT cells (NK1.1^+^CD3ε^+^). The mean values ± SD are shown (n = 3 per group in the experiment; Student’s t-test; **P<0.01). (B-C) Either PBS or γPGA was i.p. injected into WT, *lpr/lpr*, and *gld/gld* B6 mice, and splenocytes were prepared 16 hrs later. (B) Intracellular IFNγ production was analyzed in NKT cells (NK1.1^+^CD3ε^+^). The mean values ± SD are shown (n = 3 per group in the experiment; Student’s t-test; **P<0.01). Two-way ANOVA (genotype × treatment) showed an interaction between these two factors. (C) The absolute number of basophils (FcεRI^+^DX5^+^) among lineage-negative cells (CD3ε^-^CD19^-^) was determined. The mean values ± SD are shown (n = 3 per group in the experiment; Student’s t-test; **P<0.01, ***P<0.001). Two-way ANOVA (genotype × treatment) showed an interaction between these two factors. (D) Basophils (6 × 10^4^ cells/well) were cultured for 12 hrs with DCs (1.2 × 10^6^ cells/well) purified from WT B6 mice, NK cells (1.2 × 10^6^ cells/well) purified from Jα18 KO B6 mice, or iNKT cells (1.2 × 10^6^ cells/well) purified from Vα14 TCR Tg B6 mice in the presence of either PBS or γPGA (1 or 3 mg/ml). The frequency of apoptotic cells (annexin-V^+^7AAD^-^) among basophils was assessed by flow cytometric analysis. The mean values ± SD are shown (n = 3 per group in the experiment; Student’s t-test; *P<0.05, **P<0.01). Two-way ANOVA (cells × treatment) showed an interaction between these two factors (^##^P<0.01, ^###^P<0.001). (E) Basophils (6 × 10^4^ cells/well) were cultured for 12 hrs with or without DCs (1.2 × 10^6^ cells/well) purified from WT B6 mice or iNKT cells (1.2 × 10^6^ cells/well) purified from Vα14 TCR Tg B6 mice in the presence of PBS or γPGA (3 mg/ml). Neutralizing mAbs specific for IFNγ (5 μg/ml), TNFα (5 μg/ml), or IL12 (5 μg/ml) were added during the culture. The frequency of apoptotic cells (annexin-V^+^7AAD^-^) among basophils was assessed by flow cytometric analysis. The mean values ± SD are shown (n = 3 per group in the experiment). Two-way ANOVA (neutralizing Ab × treatment) showed an interaction between these two factors (^#^P<0.05, ^##^P<0.01, ^###^P<0.001). (F-G) Total splenocytes purified from WT B6 mice were cultured in the presence of either LPS (1 μg/ml) or γPGA (3 mg/ml). Intracellular IFNγ (F) and TNFα (G) production were analyzed in NK (NK1.1^+^CD3ε^-^) or NKT cells (NK1.1^+^CD3ε^+^) at the indicated time points. The mean values ± SD are shown (n = 4 per group in the experiment). (H-I) Total splenocytes purified from WT and CD1d KO B6 mice were cultured in the presence of either LPS (1 μg/ml) or γPGA (3 mg/ml). Intracellular IFNγ (H) or TNFα (I) production was analyzed in NK cell populations (NK1.1^+^CD3ε^-^) at the indicated time points. The mean values ± SD are shown (n = 4 per group in the experiment; Student’s t-test; **P<0.01, ***P<0.001).

Engagement of cell death surface receptor Fas by Fas ligand (FasL) has an important function in immune cell homeostasis and contributes to the cytotoxic activity of cytotoxic T, NK, and iNKT cells [30], and we found that administration of γPGA increased FasL expression on NKT cells compared with PBS-treated mice (Fig 4A). To examine the possibility that the reduction in basophils by γPGA is mediated through Fas/FasL-dependent responses, we utilized Fas-mutant (*lpr/lpr*) and FasL-mutant (*gld/gld*) B6 mice. Sixteen hrs after γPGA injection, NKT cells from either *lpr/lpr* or *gld/gld* B6 mice produced IFNγ in amounts comparable to WT B6 mice (Fig 4B), suggesting that NKT cell activation was not significantly affected by the blockade of Fas/FasL signaling. In addition, both the *lpr/lpr* and *gld/gld* B6 mice did not show significant differences with regard to basophil depletion by γPGA compared with WT B6 mice (Fig 4C). As Fas/FasL signaling exerts little effect on γPGA-mediated basophil reduction, we next investigated whether iNKT cell-derived cytokines such as IFNγ and TNFα induce the increased apoptosis of basophils. To test this possibility, we examined whether the addition of iNKT cells and the neutralization of iNKT cell-derived cytokines can affect the basophil apoptosis elicited by γPGA treatment. We found that the apoptotic population of basophils was synergistically increased in co-culture with iNKT cells plus DCs compared with co-culture with either iNKT cells or DCs alone in the presence of γPGA. Considering the ability of NK cells to produce proinflammatory cytokines such as IFNγ, we also examined whether NK cells have similar influence on the apoptosis of basophils. Although NK cells induced γPGA-dependent basophil apoptosis, the level of basophil apoptosis by co-culture with NK cells plus DCs was not as significant as that by co-culture with iNKT cells plus DCs. These results indicated that the activation of iNKT cells by γPGA-stimulated DCs enhanced the apoptosis of basophils at the early time points (Fig 4D). To examine the responsiveness of basophils to proinflammatory cytokines, we compared the level of cytokine receptors for IFNγ, TNFα, and IL12 expressed on basophils from both γPGA- and PBS-treated mice. Basophils from PBS-treated control mice expressed IFNγR and TNFαR but not IL12R on their surfaces. However, γPGA treatment increased the expression of all three cytokine receptors on basophils significantly much higher than those from control mice (S4 Fig). In addition, under the same conditions as those shown in Fig 4D, basophil apoptosis due to γPGA treatment was inhibited by the neutralization of IFNγ and TNFα with anti-IFNγ and anti-TNFα mAbs and synergistically inhibited by combined neutralization of both IFNγ and TNFα (Fig 4E). Taken together, these results suggest that during γPGA-mediated immune responses, homeostasis of the basophil population is largely dependent on iNKT cell-derived Th1-type cytokines such as IFNγ and TNFα rather than the Fas/FasL pathway. Here, we showed that reduction of basophils by γPGA was iNKT cell-dependent. However, to some extent, NK cells contributed to γPGA-mediated basophil reduction as shown in Fig 4D. Thus, we hypothesized that different contribution of NKT and NK cells to γPGA-mediated basophil reduction may come from their distinct kinetics of cytokine production. To test this possibility, we examined the kinetics of cytokine production in these cells following γPGA stimulation. We found that NKT cells began to produce a large amount of IFNγ and TNFα within 4–8 hrs whereas NK cells started producing these cytokines approximately 20–24 hrs after γPGA stimulation. Moreover, the percentages of IFNγ- and TNFα-producing NKT cells were higher than those of NK cells (Fig 4F and 4G). Thus, these results demonstrated that upon γPGA stimulation NKT cells exhibit much faster kinetics in cytokine production than NK cells, which suggests that NK cell activation could be dependent by iNKT cell activation. Furthermore, we examined whether NK cell activation could be affected by the absence of iNKT cells. To test this possibility, we compared the kinetics of IFNγ and TNFα production of NK cells between WT and CD1d KO B6 mice in response to γPGA stimulation. We found that cytokine production by CD1d KO NK cells was significantly lower than that of WT NK cells within 20–32 hrs after γPGA stimulation (Fig 4H and 4I), indicating that optimal NK cell activation by γPGA requires iNKT cells.

### iNKT cells are required for inhibition of papain-induced basophil-specific Th2 differentiation upon γPGA treatment

**Fig 5:**
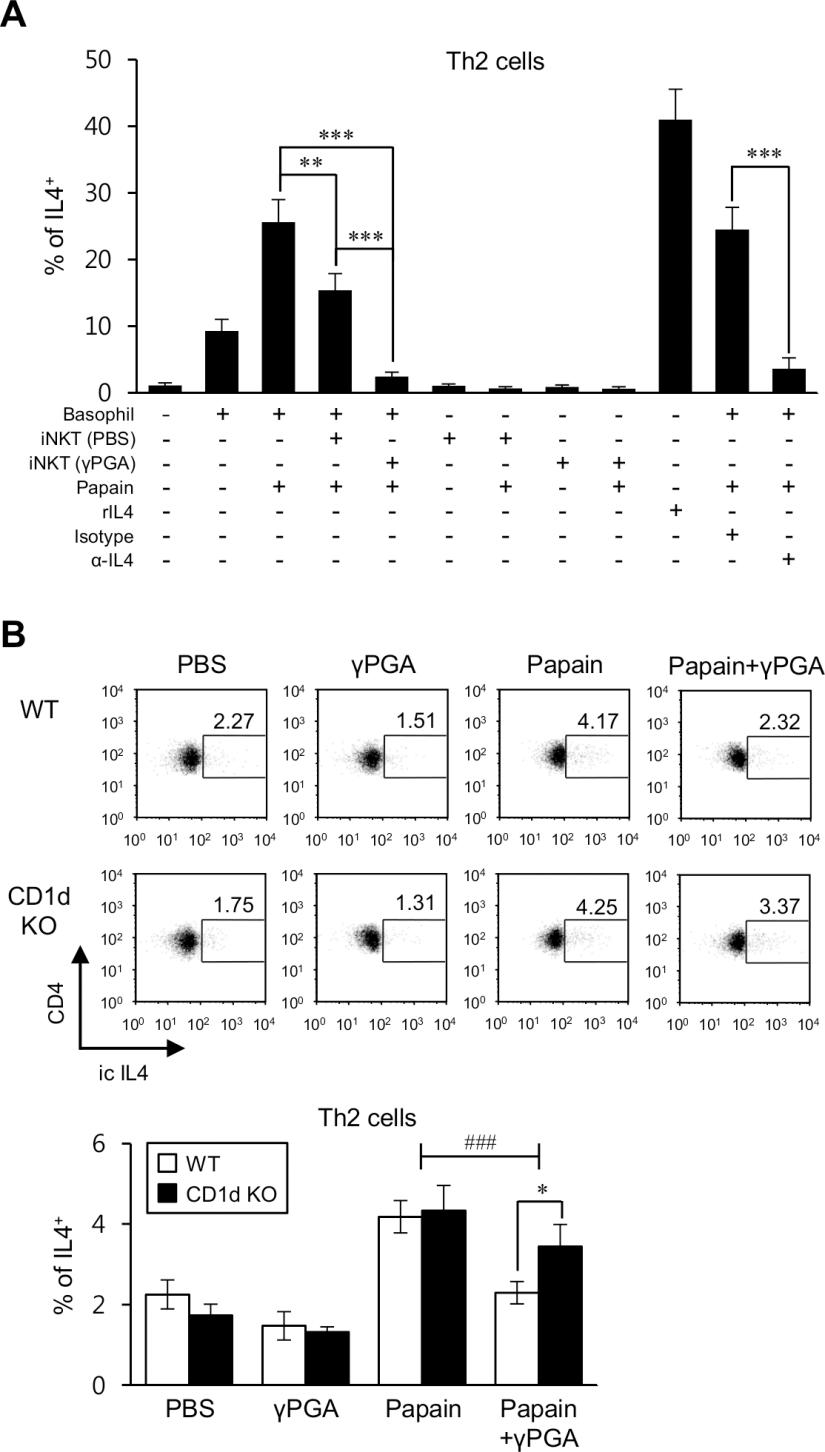
iNKT cells are required for inhibition of papain-induced basophil-specific Th2 differentiation upon γPGA treatment. (A) Naive CD4^+^CD62L^+^ T cells were cultured with a combination of basophils (6 × 10^4^ cells/well), papain (25 μg/ml), rIL4 (10 ng/ml), or neutralizing anti-IL4 mAbs (5 μg/ml) in the absence or presence of iNKT cells (1.2 × 10^6^ cells/well) purified from Vα14 TCR Tg B6 mice treated i.p. with either PBS or γPGA for 5 days. Intracellular IL4 production was analyzed in CD4^+^ T cells. The mean values ± SD are shown (n = 3 per group in the experiment; Student’s t-test; **P<0.01, ***P<0.001). (B) WT and CD1d KO B6 mice were immunized i.p. with 500 μg papain once a week for 2 weeks. Either PBS or γPGA (2 mg) was i.p. injected into PBS or papain-treated mice a total of 6 times during 2 weeks. Intracellular IL4 production in CD4^+^ T cells (CD3ε^+^CD4^+^) was assessed via flow cytometry on day 14 after the first immunization. The mean values ± SD are shown (n = 3 per group in the experiment; Student’s t-test; *P<0.05). Two-way ANOVA (genotype × treatment) showed an interaction between these two factors (^###^P<0.001).

As shown in Fig 1, we confirmed that IL4 produced by papain-stimulated basophils was involved in the Th2 differentiation as previously described [9]. Next, to investigate directly whether iNKT cells activated by γPGA have inhibitory influence on papain-induced basophil-specific Th2 differentiation, naive CD4^+^ T cells were co-cultured with basophils and iNKT cells purified from either PBS- or γPGA-treated mice. The Th2 differentiation by basophils was significantly decreased in co-culture with iNKT cells from γPGA-treated mice compared with co-culture with iNKT cells from PBS-treated mice (Fig 5A). We found that papain-induced IL4 production by Th2 cells was almost completely abrogated upon γPGA stimulation in WT B6 mice but was only partially affected in CD1d KO B6 mice (Fig 5B). Two-way ANOVA analysis on papain-injected groups of mice showed a significant interaction between γPGA treatment and genotype (presence or absence of iNKT cells). Taken together, these results suggest that iNKT cells activated by γPGA play critical roles in suppression of Th2 differentiation induced by papain treatment.

## Discussion

In this study, we demonstrate that γPGA treatment leads to an increase in apoptosis of the basophil population via the TLR4/DC/IL12 axis, a process that is mediated by iNKT cells producing Th1-type cytokines such as IFNγ and TNFα.

As iNKT cells can produce a large amount of cytokines at early time points during immune responses, these cells are thought to be key regulators determining the type of immune response. In addition, iNKT cells produce either Th1 (e.g., TNFα, IFNγ, and IL2) or Th2 cytokines (e.g., IL4, IL5, IL10, and IL13) depending on the type of stimulant, often both cytokines. For example, although α-GalCer, a well-known iNKT cell agonist, stimulates iNKT cells to produce both IL4 and IFNγ, OCH and α-C-Gal, derivatives of α-GalCer, activate iNKT cells to secrete IL4 and IFNγ, respectively [11, 31, 32]. Thus, selection of the appropriate glycolipid antigens for iNKT cell activation is required to achieve (induce) the desired immune responses for therapeutic purposes in the treatment of basophil-mediated Th2 allergic immune responses, such as asthma.

TLR4 is known to promote apoptosis in some cell types, including pancreatic cells and microglia [33, 34], and basophils constitutively express TLR4 on their cell surface as shown in S2 Fig. Thus, we addressed whether γPGA might directly promote basophil apoptosis via the TLR4 pathway. However, our *in vitro* and *in vivo* data (Fig 2G; Fig 4D) reveal no direct activation or sign of apoptosis in basophils, suggesting that γPGA did not induce basophils to undergo the apoptotic pathway via TLR4 expressed on their surfaces.

In addition, it has been demonstrated that the dysregulated expansion of basophils induces a shift from Th1 to Th2 responses [26, 35], whereas Th1 transcription factor STAT1 expression inhibits IL4 production by basophils [25]. Because basophils are one of the key regulators determining the polarity of immune responses, elucidating how these cells are activated or inhibited will provide the rationale for designing promising therapeutics suitable for not only allergic diseases but also Th1-mediated autoimmune diseases such as experimental autoimmune encephalomyelitis (EAE), rheumatoid arthritis (RA), inflammatory bowel disease (IBD), and type I diabetes. Recently, we found that the suppression of EAE pathogenesis is associated with an increase in the basophil population and in IL4 production [36]. In addition, some studies have demonstrated the regulatory roles of basophils. For example, the depletion of basophils exacerbated colitis in mice due to an increase in Th1 cytokine expression [37] and the anti-FcεR1 activation of basophils delayed the onset of type I diabetes in NOD mice [38]. Moreover, our recent study showed that the RA-promoting cytokine IL32γ can activate iNKT cells in an IL12-dependent manner, leading to IFNγ production [39]. Thus, aggravation of RA by proinflammatory IL32γ treatment might be attributed to IFNγ production by iNKT cells because IFNγ is critically involved in basophil reduction. Cytokine IL3 plays a critical role in the rapid and specific expansion of basophils [40] and is known to be the unique ligand that confers protection to basophils from apoptosis [41, 42]. Intriguingly, one study has demonstrated that the IL3 autocrine loop enhances IL3 production by basophils upon IgE-dependent activation [43]. Thus, the γPGA-mediated suppression of basophils via iNKT cell activation might be a negative effect on the action of IL3, which is responsible for allergic responses. It will be worthwhile to investigate whether γPGA treatment regulates IL3 expression in basophils.

In addition to basophils, mast cells and eosinophils are also important effector cells in allergic diseases [44]. The IFNγ produced by γPGA-activated iNKT cells might act as a negative regulator in both mast cells and eosinophils, as previous studies have shown that cytokine IFNγ induces Bax- and p53-dependent apoptosis in mast cells [45] and induces not only FasL-mediated apoptosis but also inhibition of differentiation in eosinophils [46, 47]. Despite the fact that basophils and mast cells overlap in their effector functions, they are distinct cell types with regard to a variety of characteristics, including survival factors, lifespan, and development pathway [48]. Thus, further investigations are warranted to assess the effect of γPGA on Th2-type innate immune cells such as mast cells.

A new Th2-type innate immune cell called type 2 innate lymphoid cells (ILC2s) was recently identified, and emerging evidence revealed that ILC2s are lineage-negative lymphocytes implicated in the development of allergic disorders and respiratory illnesses such as asthma [49]. Studies have reported that basophil-derived IL4 enhances the expression of ILC2-derived cytokines (i.e., IL5, IL9, and IL13) and chemokines such as CCL11 in lung eosinophilia [50] and also promotes the accumulation of IL4Rα-expressing ILC2s through a TSLP-dependent immune response in the inflamed skin [51]. Because IL4 and IFNγ are known to be reciprocally antagonistic, it can be speculated that the γPGA/iNKT/IFNγ axis could modulate the activation of ILC2s, possibly by counteracting basophil-derived IL4. Thus, it is important to examine the effects of γPGA on the function of ILC2s in the future studies.

Because a single i.p. injection of γPGA has depleting effect on basophils, we wondered how long this effect can last. We observed that the reduction of the basophil population by a single γPGA injection was nearly recovered to the untreated level at 5 days post-injection. Interestingly, this recovery of basophils was inhibited by repeated *in vivo* injection of γPGA (e.g., total three injections at 48 hr intervals), suggesting that repeated γPGA injection causes regenerating basophils to undergo the apoptotic pathway. Thus, by maintaining low levels of the basophil population, the basophil-depleting effects of repeated γPGA administration might be useful in developing therapeutics for allergic diseases. Noti *et al*. have recently shown that the TSLP-elicited basophil response plays a critical role in antigen-induced food allergy [52]. As repeated oral administration of γPGA also induces basophil reduction comparable to the level of i.p. injection, the inhibitory action of γPGA on basophils also might be effective for food allergies.

In contrast to the previous studies that papain-induced Th2 responses are dependent on basophil-derived IL4 and also altered number of basophils affects either Th1/Th2 ratio or sensitivity against inflammatory diseases [8, 50], Ohnmacht *et al*. demonstrated that basophils are dispensable to induce an optimal Th2 response in response to papain stimulation [53]. Moreover, non-requirement of basophils in papain-induced Th2 responses was demonstrated with transgenic mice in which basophils were constitutively deficient using Cre/loxP system. However, constitutive deficiency of basophils in transgenic mouse model using Cre/loxP system might be different from other models in which basophils were inducibly depleted using DT or basophil-depleting antibody during immune responses, which could generate distinct outcome. In case of our experimental setting, consistent with the previous studies showing basophil dependency in papain-induced Th2 responses, our results showed that papain injection induced not only basophil accumulation but also an increase in Th2 cells in the spleen.

We demonstrated that γPGA-mediated suppression on papain-induced Th2 differentiation was significantly dependent on the activation of iNKT cells. These results could be explained by different kinetics of cytokine production between iNKT and NK cells. Basophil apoptosis is highly dependent on iNKT cells at early time point, for example, 16 hrs post γPGA treatment. However, at later time points (i.e., after 16 hrs post-γPGA stimulation), other IFNγ-producing cells such as NK and Th1 cells in addition to iNKT cells might act as the inhibitors of papain-induced basophil-specific Th2 differentiation.

Taken together, we demonstrate for the first time that iNKT cells are one of the key regulators of basophils and furthermore that iNKT cell-derived IFNγ is a potent inhibitory cytokine in basophil survival and in the suppression of the basophil-mediated Th2 response. These mechanisms may represent a therapeutic strategy to protect against the onset of allergic diseases.

## Supporting Information

S1 Fig Comparison of IL12 receptor expression between NK and NKT cells. Splenocytes were prepared from WT B6 mice. Expression of IL12 receptor on NK (CD3ε^-^NK1.1^+^) and NKT cells (CD3ε^+^NK1.1^+^) was assessed by flow cytometry. The mean values ± SD are shown (n = 3 per group in the experiment; Student’s t-test; ***P<0.001).
(TIFF)

S2 Fig The surface expression of TLR4 on basophils. Splenocytes were prepared from WT B6 mice. Expression of TLR4 on DCs, macrophages, and basophils was assessed by flow cytometry. The mean values ± SD are shown (n = 3 per group in the experiment; Student’s t-test; ***P<0.001).
(TIFF)

S3 Fig Comparison of the basophil reduction between mice treated either i.p. or orally with γPGA. (Fig A) WT B6 mice were treated either i.p. or orally with γPGA (2 mg) 3 times for 5 days. (Fig B) The absolute number of basophils in mice treated either i.p. (left panel) or orally (right panel) was assessed by flow cytometry. The mean values ± SD are shown (n = 3 per group in the experiment; Student’s t-test; ***P<0.001).
(TIFF)

S4 Fig Surface expression of cytokine receptors to IFNγ, TNFα, and IL12 on basophils. Splenocytes were prepared from WT B6 mice. The expression of cytokine receptors to IFNγ, TNFα, and IL12 on basophils was assessed by flow cytometric analysis. The mean values ± SD are shown (n = 3 per group in the experiment; Student’s t-test; **P<0.01, ***P<0.001).
(TIFF)
