# Supplementary figures and images for: iNKT Cells Are Responsible for the Apoptotic Reduction of Basophils That Mediate Th2 Immune Responses Elicited by Papain in Mice Following γPGA Stimulation
Source: PLoS One. 2016 Apr 6;11(4):e0152189. doi: 10.1371/journal.pone.0152189 (PMC4822947; doi:10.1371/journal.pone.0152189)

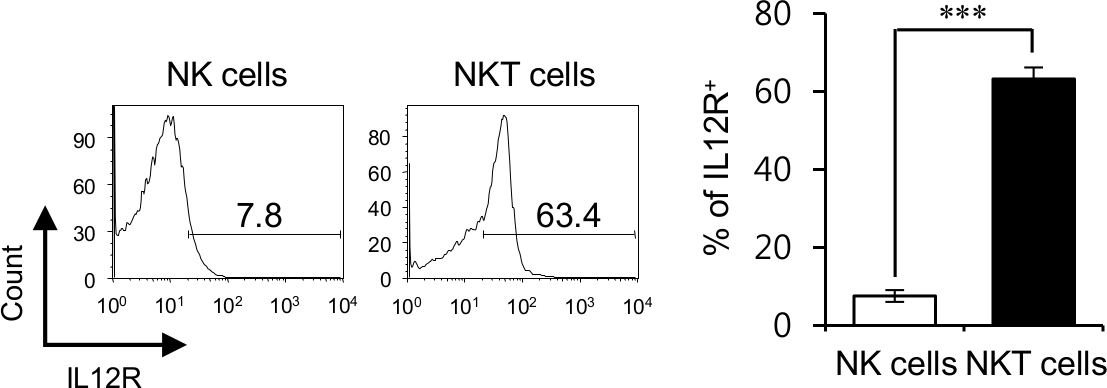

Supplement: S1 Fig — Splenocytes were prepared from WT B6 mice. Expression of IL12 receptor on NK (CD3ε-NK1.1+) and NKT cells (CD3ε+NK1.1+) was assessed by flow cytometry. The mean values ± SD are shown (n = 3 per group in the experiment; Student’s t-test; ***P<0.001). (TIFF) [file pone.0152189.s001.TIFF]

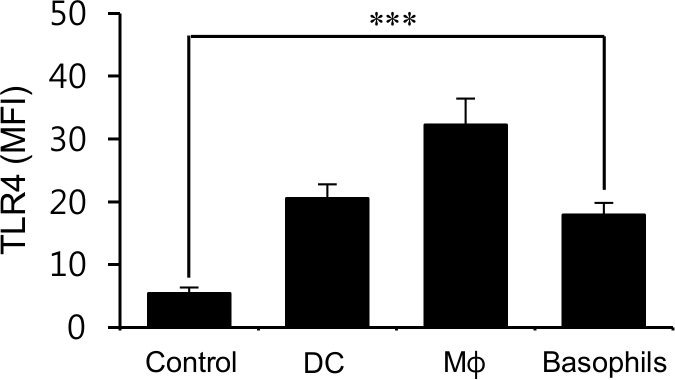

Supplement: S2 Fig — Splenocytes were prepared from WT B6 mice. Expression of TLR4 on DCs, macrophages, and basophils was assessed by flow cytometry. The mean values ± SD are shown (n = 3 per group in the experiment; Student’s t-test; ***P<0.001). (TIFF) [file pone.0152189.s002.TIFF]

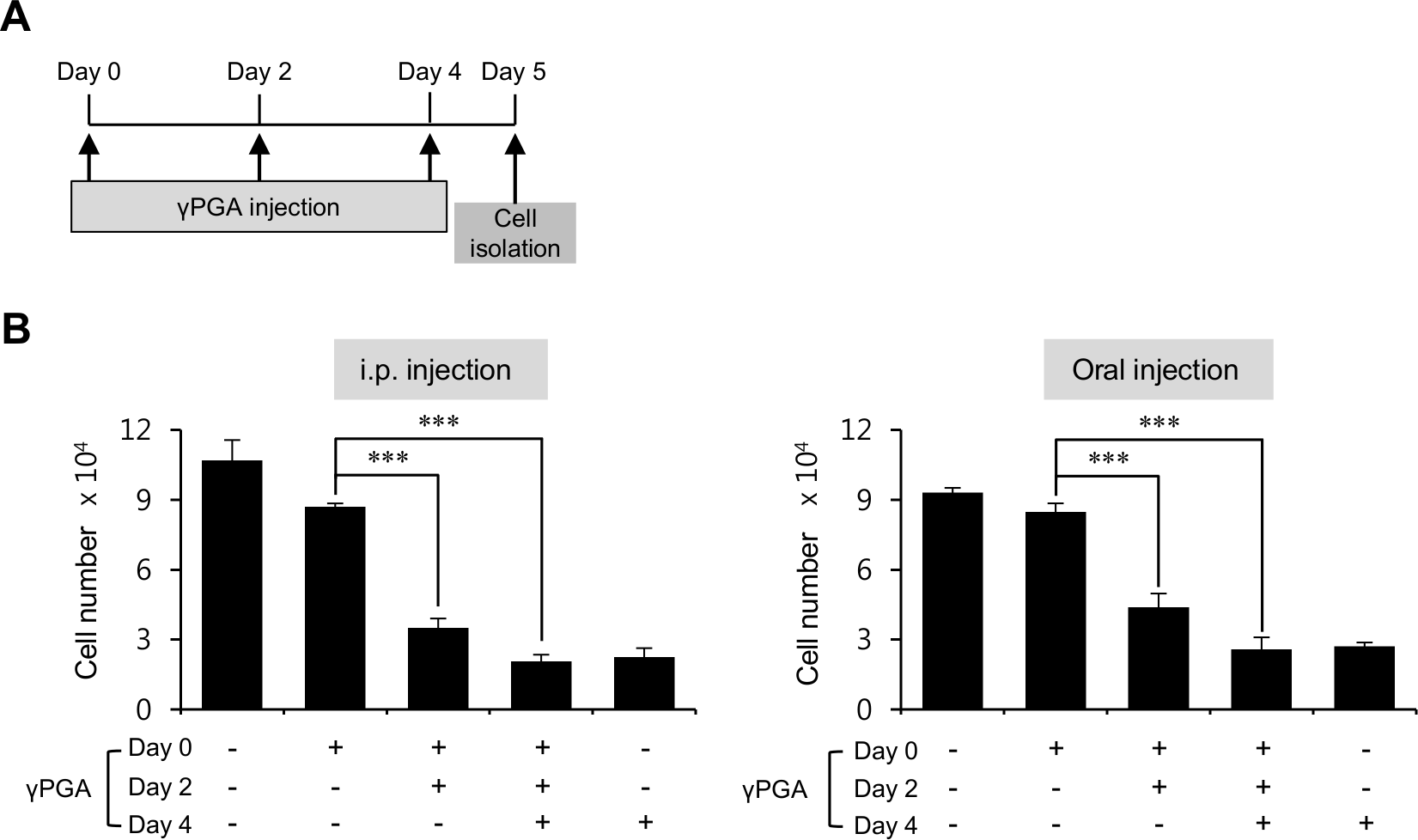

Supplement: S3 Fig — (Fig A) WT B6 mice were treated either i.p. or orally with γPGA (2 mg) 3 times for 5 days. (Fig B) The absolute number of basophils in mice treated either i.p. (left panel) or orally (right panel) was assessed by flow cytometry. The mean values ± SD are shown (n = 3 per group in the experiment; Student’s t-test; ***P<0.001). (TIFF) [file pone.0152189.s003.TIFF]

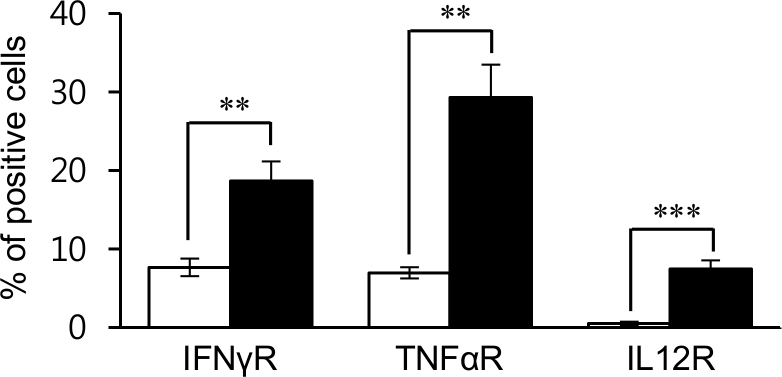

Supplement: S4 Fig — Splenocytes were prepared from WT B6 mice. The expression of cytokine receptors to IFNγ, TNFα, and IL12 on basophils was assessed by flow cytometric analysis. The mean values ± SD are shown (n = 3 per group in the experiment; Student’s t-test; **P<0.01, ***P<0.001). (TIFF) [file pone.0152189.s004.TIFF]
